# Effect of Addition Levels of By-Product Mixture (Apple Pomace: Red Potato Pulp: Sugar Beet Pulp) on Phytochemical Profile, Antioxidant Activity and Physical Properties of Extruded Corn Snacks

**DOI:** 10.3390/molecules31061037

**Published:** 2026-03-20

**Authors:** Rafał Ziobro, Dorota Gumul, Renata Sabat, Anna Wywrocka-Gurgul, Tomasz Zięba

**Affiliations:** 1 Department of Carbohydrate Technology and Cereal Processing, Faculty of Food Technology, University of Agriculture in Krakow, 122 Balicka Street, 30-149 Krakow, Poland; rafal.ziobro@urk.edu.pl (R.Z.); renata.sabat@urk.edu.pl (R.S.); anna.wywrocka-gurgul@urk.edu.pl (A.W.-G.); 2 Institute of Sport, Tourism and Nutrition, Faculty of Biological Sciences, University of Zielona Góra, 65-417 Zielona Góra, Poland; tomasz.zieba@upwr.edu.pl; 3 Department of Food Storage and Technology, The Faculty of Life Sciences and Technology, Wrocław University of Environmental and Life Sciences, Chełmońskiego 37, 51-630 Wrocław, Poland

**Keywords:** by-products, snacks, phytochemical compounds, dietary fiber, nutritional composition, food quality

## Abstract

Plant by-products such as apple pomace, potato pulp, and sugar beet pulp can be an excellent source of polyphenols, other phytochemicals and fiber, which is why they can be an excellent addition to snacks. Snacks, on the other hand, contain a lot of sugar and starch, which increases the risk of metabolic diseases and is unfavorable for diabetics, but after adding the above-mentioned by-products, their nutritional and health-promoting value increases. The aim of the study was to examine the effect of different addition levels of a mixture of by-products on the nutritional composition, phytochemical content, antioxidant activity, and quality of corn snacks. It was found that mixtures of by-products are an excellent addition to corn snacks in order to increase the health benefits of the product, as this additive increases the content of polyphenols, phenolic acids, anthocyanins, dietary fiber, tocopherols, especially α- and γ-tocopherol, as well as phytosterols, including β-sitosterol, stigmasterol, and campesterol. Furthermore, it increases the antioxidant potential of the snacks and the nutritional value of the final product, especially protein and ash content, reducing the starch content. Snacks containing a 20% by-product mixture may be recommended due to their improved nutritional and health-promoting properties and acceptable physical characteristics.

## 1. Introduction

Extrudates based on cornmeal are often used as snacks or ready-to-eat products (RTE) and contain high amounts of sugars and starch, which detrimentally affect their nutritional quality. They have a high glycemic index and can increase the risk of metabolic problems, such as cardiovascular disease, obesity, and type 2 diabetes [1,2]. According to the NOVA classification [3], snacks are usually classified as ultra-processed food, which is generally not preferred by dietitians. This problem could be solved by the addition of by-products with high content of dietary fiber (DF), which is often accompanied by polyphenols, and therefore could improve the nutritional quality and pro-health properties of cereal-based snacks. The above mentioned constituents (DF and polyphenols) play a chemo-preventive role in human nutrition, because they exhibit anti-radical anti-carcinogenic, anti-bacterial, anti-viral, anti-inflammatory, as well as hypoglycemic and hypotensive activity and diminish the risk of diseases such as diabetes, atherosclerosis and other cardiovascular diseases, genetic damage, degenerative bone changes, and neurodegenerative diseases, including Alzheimer’s disease [4,5,6,7].

Plant by-products are an excellent source of these compounds, as they contain a wide spectrum of phytochemicals, e.g., phenolic compounds with highly valuable antioxidant properties and dietary fiber [2,8]. Phenolic compounds are present in the cell walls of plant material and can be an integral part of dietary fiber [9,10]. Therefore, by-products such as apple pomace, red potato pulp, and sugar beet pulp constitute a combination of many health-promoting compounds that can be successfully used in the production of health-promoting corn snacks [11,12,13,14,15,16,17].

Many authors [18,19,20] consider potatoes to be a functional food due to their polyphenol content. Among the polyphenols in potatoes, phenolic acids are the most abundant group found in potato tubers. The dominant phenolic acid is chlorogenic acid, followed by its derivatives, neo- and crypto-chlorogenic acids [11,12,13]. According to Rytel et al. [13], potatoes with colored flesh are richest in chlorogenic and neochlorogenic acids, while other acids are present in smaller amounts. Akyol et al. [21] and Mäder et al. [22] also noted that potatoes with different flesh colors are characterized by low levels of phenolic acids, such as caffeic, coumaric, ferulic, sinapic, and gallic acids. Another important group of polyphenolic compounds in potatoes is flavonoids, including catechin, epicatechin, kaempferol, and rutin [11]. Anthocyanins, a subgroup of flavonoids, are found only in red and purple potatoes and/or their skins, in concentrations ranging from 5.5 to 35 mg/100 g [12]. These anthocyanins are acylated with phenolic acids, mainly ferulic and p-coumaric acids. Purple potatoes contain petunidin and malvidin 3-rutinose-5-glucoside, acylated with p-coumaric and ferulic acids, while red potatoes are rich in pelargonidin and peonidin 3-rutinose-5-glucoside, also acylated with p-coumaric and ferulic acids [23].

Apple pomace is another good source of polyphenols and fiber. Polyphenols occur in soluble form or can be bound to cell wall structures [24]. The total polyphenol content measured using the Folin–Ciocalteu reagent in apples from 40 varieties was between 66.2 and 211.9 mg/100 g [25]. This corresponds to a total polyphenol content in dried apple pomace, which is estimated between 262 and 856 mg/100 g [26,27]. The increase in polyphenol content compared to fresh apples is related to water loss during the juice pressing and apple pomace drying processes. It should also be emphasized that some of the polyphenols are transferred to the juice during apple pressing (3–10%), and the largest source of these components is the skin, seeds, and stems of apples, which constitute fractions of apple pomace. The total polyphenol content in apple pomace is also influenced by enzymes released from vacuoles during juice pressing, such as polyphenol oxidases and peroxidases, which lead to the oxidation of polyphenols and thus to a reduction in their content [26]. It is estimated that the highest concentration of phenolic compounds could be found in seeds and petioles, 3593–23,607 and 6747–59,335 mg/kg d.m., respectively, and the lowest in pulp, 1655–5314 mg/kg d.m. The dominant polyphenol in the flesh fraction of apple pomace is chlorogenic acid (405–2299 mg/kg dry weight), while in seeds and stalks it is phloridzin (2899–19,600 and 3018–40,488 mg/kg dry weight, respectively). In addition to chlorogenic acid, apple pomace pulp is also a rich source of the following flavonol glycosides: quercetin-3-O-galactoside, quercetin-3-O-glucoside, quercetin-3-O-xyloside, quercetin-3-O-arabinoside, and quercetin-3-O-rhamnoside. The content of these flavonols ranges from 27 to 579 mg/kg d.m. Flavonol glycosides are present in seeds at significantly lower levels than in other fractions of apple pomace, but the highest content of these compounds is found in petioles—84–3668 mg/kg d.m. Caffeic acid and procyanidins B2 occur at similar levels in all fractions of apple pomace, in amounts ranging from 8 to 555 mg/kg d.m. [14,15,16,24].

The main polysaccharides of the dietary fiber present in sugar beet pulp are alkali-soluble polysaccharides (ASP), consisting of arabinans, arabinogalactans, and galactans attached as side chains to pectin and partially bound to cellulose. Bioactive compounds from the polyphenol group are also partially bound to DF. These include hydroxy derivatives of cinnamic acids (e.g., ferulic acid) attached to arabinogalactans present in sugar beet [17], similarly to those found in wheat arabinoxylans [28,29]. In general, ferulic acid derivatives constitute a group of polyphenols that most often bind to fiber through covalent bonds (ester, carbon-carbon ether) [30]. In contrast, the connection between proanthocyanidins and fiber is usually formed through hydrogen bonds, hydrophobic effects, van der Waals forces, or other non-covalent interactions [31].

The above-mentioned plant by-products are an excellent source of a wide range of bioactive compounds belonging to the group of polyphenols and dietary fiber, which is why supplementation of cereal snacks with these compounds could be achieved by their addition. It should also be emphasized that extrusion is a technique that enables the utilization and full use of by-products, allowing the production of cereal snacks with enriching additives with proven health benefits [32]. The study attempted to obtain this type of snack using a mixture of the above-mentioned by-products to better utilize their unique composition. The aim of the study was to analyze the effect of different levels of a mixture of by-products on the nutritional value and content of polyphenolic compounds, tocopherols, and phytosterols in cereal snacks. In addition, the antioxidant activity of such snacks was estimated, and the functional and physical properties of the final products were characterized.

## 2. Results and Discussion

### 2.1. Characteristics of the Snack Enriching Mixture (Apple Pomace, Red Potato Pulp, Sugar Beet Pulp in a Ratio 1:1:1)

Considering the nutritional composition of the applied mixture of by-products consisting of apple pomace, sugar beet pulp, and red potato pulp in a ratio of 1:1:1, it was found that the level of dietary fiber exceeded all other compounds (Table 1). This is a logical consequence of the fact that the above-mentioned by-products are an excellent source of dietary fiber. It is especially true for apple pomace, where its content reaches 60% [33]. It should be emphasized that the determined dietary fiber consists of 2/3 insoluble fraction (IDF) and 1/3 soluble fraction (SDF). The ratio between the soluble and insoluble fractions of fiber, which is 1:2, is beneficial in terms of the health-promoting properties. Dietary fiber is characterized by hypocholesterolemic, hypoglycemic, and anticancer properties, and epidemiological studies clearly show a correlation between a decrease in dietary fiber consumption and an increase in the incidence of digestive system diseases, an increased risk of hypercholesterolemia and colon cancer [34,35]. Another very important component was total sugars, which amounted to 26 g/100 g d.m. (Table 1). This is the result of a much higher content of sugars in beet pulp, while the protein content fluctuated around 5.24 g/100 g d.m. (Table 1). It should be noted that protein from potato pulp is a complete protein with high biological value [36], which is very important when enriching grain feed mixtures with potato pulp, as grains are characterized by many limiting amino acids, including lysine. The high ash content is due to the relatively high ash content in beet pulp and potato pulp, and the fat content fluctuates around 1.47 g/100 g d.m., which is mainly related to the fat from apple pomace (Table 1).

When considering the content of polyphenolic compounds, it should be observed that the level of polyphenols is significant, but flavonoids are the dominant group among them (Table 1), as the content of flavonoids is significant in each of these three by-products [11,15,23]. Next are phenolic acids and anthocyanins, which mainly come from red potato pulp [11,21,23]. Such a high polyphenol content results in very high antioxidant activity, as determined by two independent methods using the ABTS and DPPH free radicals (Table 1).

In the case of tocopherols in the discussed by-product mixture, three times more α-tocopherol was found than γ-tocopherol. This fact is related to the fact that apple pomace and red potato pulp are sources of α-tocopherol [37,38,39]. Among phytosterols, a significant amount of β-sitosterol was noted, followed by stigmasterol and campesterol, as all components of the by-product mixture are rich in these compounds [39,40].

In summary, it should be noted that the nutritional and health-promoting value of a mixture of apple pomace, sugar beet pulp, and potato pulp (1:1:1) is high. It could be described as a concentrate of dietary fiber and polyphenols, with high levels of total sugars, protein, and ash, which is why it can be successfully used as an ingredient to enrich corn snacks, which have low dietary value.

### 2.2. Chemical Characteristics of Corn Snacks Produced with an Enriching Mixture of By-Products

#### 2.2.1. Composition of Snacks with a Share of a Mixture of By-Products

The chemical composition of the snacks obtained with the addition of a mixture of by-products is noteworthy, as it reduces the content of an undesirable ingredient in these snacks, namely starch (Table 2). After adding 10% of the mixture, the amount of starch decreases by 6%, and after adding 30%, the starch content decreases by 23% in enriched snacks compared to the control (Table 2). A three- to six-fold increase in total sugars was also observed in snacks enriched with a mixture of by-products compared to snacks without it (Table 2). The observed increase in sugars, mainly from apple pomace, however, is compensated by a far greater increase in the soluble and insoluble fiber fraction, the source of which is the by-products present in the added mixture. The levels of both soluble and insoluble fiber in snacks containing a mixture of by-products were significantly elevated compared to the control. The change in the insoluble fiber fraction ranged from a 6- to 25-fold increase in this component in snacks containing a mixture of by-products compared to the control. In contrast, in snacks containing by-products, a 4- to 19-fold increase in the soluble fiber fraction was observed compared to the control. The total fiber (TDF) content ranged from 2.69 to 11.67 g/100 g of dry matter, which was 5 to 22.4 times higher in enriched snacks compared to the control of 0.52 g/100 g d.m. (Table 2), which is a logical consequence of the use of by-products that are a concentrate of dietary fiber.

Dietary fiber is a chemically heterogeneous complex consisting of insoluble and soluble fractions, each of which has a different physiological effect. Insoluble fiber is recommended for the prevention and treatment of colon diseases such as chronic constipation, irritable bowel syndrome, hemorrhoids, and diverticulosis. Soluble fiber, on the other hand, has hypocholesterolemic, hypoglycemic, and anticancer properties. The main sources of fiber in the diet are grains, but above all vegetables and fruits, especially by-products such as pomace and pulp [2,41,42]. The introduction of by-products rich in the earlier-mentioned polyphenolic compounds and dietary fiber is therefore highly justified to obtain snacks with high nutritional value. An increase in protein and ash content could also be noticed in snacks containing by-products compared to the control, in contrast to the constant fat content (Table 2). The increase in protein content by as much as 17% with a 30% addition of a mixture of by-products to snacks (Table 2) is mainly due to the use of potato pulp. In this regard, it should be emphasized that potato protein has high biological and nutritional value because it contains all essential amino acids (such as lysine, lecithin, phenylalanine, tyrosine) that are not synthesized by the human body. The biological value of potato protein is comparable to that of chicken egg protein (chemical score CS is in the range of 57–69%) [36] and other animal proteins and is significantly higher than the biological value of proteins from peas, wheat, and rice. In addition, potato protein is the best source of lysine in the plant world [36]. The increase in ash content (Table 2) in snacks is due to the use of a mixture of by-products, especially apple pomace and beet pulp [43,44], where the ash content is 1.88% and 7%, respectively.

A strong negative correlation was observed between the fat content in the analyzed snacks and individual dietary fiber fractions and total sugar content (*r* between −0.950 and −0.993). On the other hand, a strong positive correlation was found between starch and fat in the snacks (*r* = 0.958). A strong positive correlation was also noted between individual dietary fiber fractions and the content of anthocyanins, polyphenols, flavonoids and phenolic acids, as well as alpha-tocopherol in the snacks (*r* between 0.973 and 0.998; Table S1).

#### 2.2.2. Polyphenol Compounds of Snacks with a Share of a Mixture of By-Products

It was found that the inclusion of by-products contributed to an increase (2 to 7 times) in total polyphenols (TPC) in snacks containing a mixture of by-products, compared to control snacks.

It can be concluded that the increase in total polyphenol content was gradual in relation to the proportion of by-products in the snacks (Table 3). In the studies by Gumul et al. [45] on the effect of adding defatted blackcurrant seeds to extruded corn products, it was also found that the amount of TPC increased with their rising level. The study by Reis et al. [46] analyzed the effect of apple pomace on extruded products. It was found that a 10% addition of pomace resulted in a 73% increase in total polyphenol content compared to unenriched snacks, and a 20% and 30% addition resulted in an increase of 37% and 82%, respectively, compared to the control [46]. In a study by Korkerd et al. [47], the effect of adding food processing by-products (defatted soybean meal, germinated brown rice flour, and mango peel fiber) to extruded snacks was investigated. It was noted that the by-products increased the polyphenol content in the snacks from 0.3 to 6.7–10.06 mg GAE/g of snack, depending on the extrusion conditions [47]. In studies by Oniszczuk et al. [48] on the effect of adding elderberry flowers and fruits to corn snacks, it was also proven that the polyphenol content in snacks increased proportionally with the addition of elderberry. Gumul et al. [49] came to similar conclusions when studying the effect of fortifying corn snacks with red and purple freeze-dried potatoes, as the polyphenol content in the snacks increased with the increasing proportion of potatoes in the mixture. A 5% addition of red potatoes to snack formulation resulted in a 25% increase in polyphenol content compared to control corn snacks. However, with 15% and 25% red potatoes in the enriched snacks, the polyphenol content increased by 99% and 188%, respectively, compared to the control [49]. Similar observations were made in studies by Drożdż et al. [50] on snacks containing currant and chokeberry pomace, where an increase in total polyphenols was observed in enriched snacks compared to corn extrudates. A 10% addition of chokeberry pomace resulted in a 44-fold increase in total polyphenol content compared to the control sample. Apart from that, a 15% and 20% addition of chokeberry to the crisps resulted in a 120-fold and 178-fold increase in total polyphenols, respectively, compared to the control. In a study by Bekele et al. [51], the effect of the composition of a chickpea and sorghum mixture on the polyphenol content in finished products—expanded snacks—was investigated. It was found that the content of phenolic compounds increased with the increase in the proportion of sorghum in the mixture. In a study by Yagci et al. [52], extruded snacks enriched with tomato pomace powder were examined. As expected, the phenolic content in the control sample was significantly lower than in snacks with added tomato pomace. With a 5% addition of tomato pomace, the total polyphenol content increased twofold compared to the control sample. In contrast, 10% and 15% tomato pomace content resulted in a 3.5- and 5-fold increase in total polyphenol content, respectively, compared to corn snacks [52]. In a study by Promsakha na Sakon Nakhon et al. [53] on the effect of adding pumpkin powder to corn snacks, it was found that as the proportion of pumpkin flour increased, so did the polyphenol content.

The snacks produced with a mixture of by-products revealed high levels of flavonoids in comparison to the control (Table 3). Even the smallest—10% share of by-products contributed to a 5-fold increase in their amount as compared to non-enriched snacks. The application of 20 and 30% share of pomace/pulp mixture in snack formulation caused a 13- and 21-fold increase in flavonoid content compared to the control sample (Table 3). A similar increase was observed for phenolic acids and flavonols in extrudates with the above-mentioned additives, which were 2–7.5 times and 6–23 times higher than in the control, respectively (Table 3). In the case of anthocyanins, which appeared in snacks due to the presence of red potato pulp in the enrichment mixture, a significant amount of 1.05–10.2 mg/100 g d.m. was also observed (Table 3).

In general, it was found that as the level of a mixture of by-products increased, so did the level of flavonoids, polyphenols, phenolic acids, flavonols, and anthocyanins, although this increase was not proportional to the amount of additive introduced, but many times greater. This can be explained by the various changes that occur during the extrusion process, including isomerization and the release of flavonoids and phenolic acids in particular from glycosidic and ester bonds, thanks to which they are more easily extracted during final assessment, hence their apparent quantity in the analyzed test material is high [49].

In a study by Oniszczuk et al. [48] on the effect of adding elderberry flowers and elderberries to corn snacks, it was found that the flavonoid content in the snacks increased proportionally with the addition of elderberries. Fortifying snacks with 5, 10, and 20% elderberry fruit resulted in an increase in flavonoid content of 30, 157, and 359%, respectively, compared to corn snacks. A 5% and 10% share of flowers in snacks resulted in a decrease in flavonoid content by 30% and 157%, respectively, compared to corn snacks. However, a 20% addition of flowers resulted in a more than threefold increase in flavonoid content compared to the control sample [48]. In studies by Gumul et al. [49] on the effect of adding red potatoes to corn snacks, an increase in flavonoid content was observed with a rising level of potato addition. A 5% share of red potatoes in snack formulation resulted in a 140% increase in flavonoid content compared to corn snacks (control). In contrast, 15% and 25% introduction of red potatoes in snacks resulted in a 387% and 484% increase in flavonoid content, respectively, compared to the control sample.

In the study by Drożdż et al. [50], the effect of adding currant and chokeberry pomace on the phenolic acid content in corn snacks was investigated. An increase in phenolic acid content was observed with increasing levels of fruit pomace addition. In the case of chlorogenic acid, a 10% addition of chokeberry pomace resulted in an almost 78-fold increase in its content compared to the control sample. Larger, 15% and 20% addition of chokeberry to the crisps resulted in an almost 102-fold and 154-fold increase in chlorogenic acid content, respectively, compared to the corn snacks. In the case of blackcurrant pomace, a 10% addition of the by-products to snack formulation resulted in an almost 39.5-fold increase in chlorogenic acid content compared to the control sample. Enriching the snacks with 15% and 20% blackcurrant resulted in an almost 43-fold and over 39-fold increase in chlorogenic acid content, respectively, compared to corn snacks [50]. In another study by Reis et al. [46] on the effect of incorporating apple pomace into extruded products, it was found that as the proportion of apple pomace in snacks increased, the flavonol content also increased. A 10% and 20% addition of apple pomace had the same effect. Both proportions contributed to a 3.25-fold increase in flavonol content compared to corn snacks. In contrast, a 30% proportion of pomace in snacks resulted in a 4-fold increase in flavonol content compared to the control sample [46]. Arribas et al. [54] also studied the effect of adding bean meal and carob meal to rice extrudates. They observed that as the amount of carob meal added increased, the flavonol content also increased.

**Figure 1:**
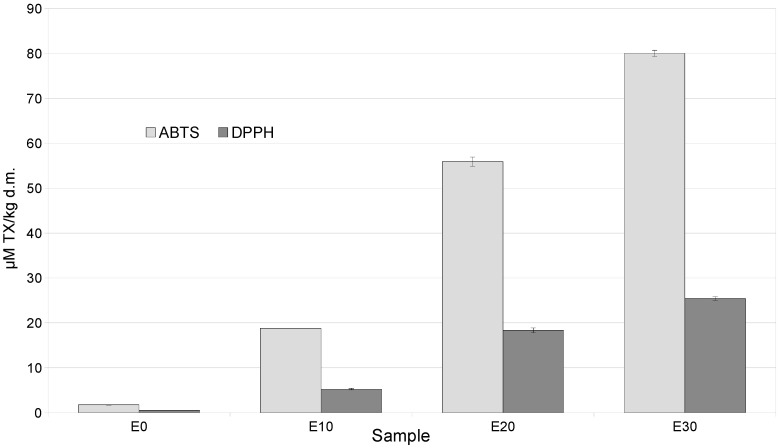
Antioxidant activities (ABTS, DPPH) of extrudates enriched with varying levels of a mixture of by-products.

The increase in polyphenols and their fractions was accompanied by an increase in antioxidant activity measured by ABTS and DPPH in snacks containing a mixture of by-products, compared to the control, by 20–80 times (ABTS) and 5–25 times (DPPH) (Figure 1).

This was confirmed by strong positive correlations between ABTS and anthocyanin content r = 0.992, total polyphenol content r = 0.983, flavonoid content r = 0.991, and phenolic acid content r = 0.999. In addition, strong positive correlations were found between DPPH and anthocyanin content at r = 0.996, total polyphenol content at r = 0.977, flavonoid content at r = 0.986, and phenolic acid content at r = 0.998. Furthermore, the antioxidant activity of the discussed snacks was influenced by tocopherols such as α-tocopherol, as the correlation between this isomer and ABTS and DPPH was r = 0.984 and r = 0.976, respectively (Table S1).

#### 2.2.3. Tocopherols in Snacks with a Share of a Mixture of By-Products

In corn snacks used for control, δ and γ tocopherols were found, but α tocopherol and β tocopherols were not detected (Table 4). This is because cornmeal is the primary source of γ tocopherol and δ tocopherol [55]. In the case of δ-tocopherol, whose content is low in both apple pomace and red potato pulp, and is not present at all in beet pulp [37,38,44], it was observed that enriching snacks with a mixture of by-products did not cause any changes in its content in snacks (Table 4).

In contrast, the content of α-tocopherol, which was not detected in the control, increased with the increase in the content of by-products in the snacks (Table 4). This results from the very high content of α-tocopherol in both apple pomace and potato pulp [37,38,39], which make up the mixture added to enrich the snacks. Similarly, in the case of γ-tocopherol, an increase in its content was observed in snacks made with a mixture of by-products compared to the control. However, when comparing the increase in α-tocopherol content in snacks enriched with γ-tocopherol, it was clearly observed that the amount of α-tocopherol is significantly higher in these snacks (Table 4) due to the fact that the dominant tocopherol in apple pomace and red potato pulp is α-tocopherol [37,38,39]. In addition, β-tocopherol, which was absent from the control (Table 4), was found in snacks enriched with by-products. It should also be noted that during extrusion, deviations in the content of tocopherols and tocotrienols may occur as a result of changes in the chemical structure of these compounds due to their thermal degradation, depolymerization, and recombination of their molecular fragments under the influence of high pressure, temperature, and shear forces prevailing in the extruder [56]. Individual isomers of tocopherols and tocotrienols will react differently; it is assumed that α and β tocopherol are the least stable, while γ tocopherol is stable [37,38,56]. In addition, Zieliński et al. [57] noted a change in the content of tocopherols and tocotrienols in rye grain extrudates at a level of 91% when they used extrusion at a temperature of 120 degrees Celsius, while at the other temperatures of 160 and 200 degrees Celsius, the reduction was 75.68%. Tiwari and Cummins [58] had a similar opinion, stating that high extrusion temperatures and short times can cause tocopherols to stabilize, hence their lower losses at higher temperatures during this process. On the other hand, Shin et al. [59] noticed significant losses of tocopherols at extrusion temperatures of 110 to 140 degrees Celsius. Nevertheless, it should be emphasized that the mixtures used to produce the snacks enriched them mainly with α-tocopherol, γ-tocopherol and β-tocopherol. The amount of these compounds in the snacks was significantly lower than the percentage of these mixtures, which is due to the changes in these compounds during extrusion. By-product mixtures did not enrich the snacks in δ-tocopherol, as its amount in corn grits is very high, while in the by-product mixture it is low.

#### 2.2.4. Phytosterols in Snacks with a Share of a Mixture of By-Products

Regarding phytosterols, it should be noted that snacks containing a mixture of by-products were characterized by a very high β-sitosterol content. The increase in β-sitosterol content after adding 10% of the mixture of by-products was twofold compared to the control, and with a 30% addition, it reached 11 times the amount of this phytosterol in relation to the control (Table 5).

Such a large increase is because the mixture of by-products is rich in these types of compounds, especially apple pomace and red potato pulp, particularly in β-sitosterol. The amount of β-sitosterol in apple pomace reaches 1147 mg/g, and in red potatoes 1.82 mg/100 g [39,40,60]. Sugar beet pulp also contains β-sitosterol, stigmaceterol, and campesterol, which are predominant in plants [61]. An increase in campesterol and stigmasterol was also observed in snacks containing a mixture of by-products compared to the control snacks. The amount of other phytosterols, δ-5 and δ-7-avenasterols and δ-7-stigmasterol, varied in these products. The content of 5-avenasterol increased from 3 times in snacks with the addition of 10% of a mixture of by-products to 22 times in snacks with the addition of 30%. The amount of δ-7-stigmasterol in snacks decreased (about 70%) with the addition of 10% of a mixture of by-products. In the snacks with 20% and 30% addition of this mixture, the amount of δ-7-stigmasterol decreased by 55% and 11%, respectively, compared to the control. The exception was δ-7-avenasterol, the amount of which remained constant both in the control and in snacks produced with a mixture of by-products (Table 5). The amount of campesterol increased in snacks using by-products 2 to 7.6 times compared to the control, and stigmasterol from 25% to 3 times (Table 5). The amount of cholesterol at a 10% by-product content was identical to the control, but a 20% and 30% addition caused an increase in cholesterol by 56% and 118%, respectively, in snacks with by-products compared to the control (Table 5).

It should be noted that extrusion causes phytosterols degradation, which increases with the temperature of the process [62]. Therefore, a temperature of 160 °C was used here, along with an appropriate selection of by-products rich in phytosterols to counteract this problem. Phytosterols are important health-promoting substances with hypocholesterolemic properties, as well as anti-inflammatory, antioxidant, and anticancer effects [63,64]. The high amount of these compounds in snacks is an important achievement of this work.

### 2.3. Physical Properties of Snacks with a Share of a Mixture of By-Products

Table 6 represents the physical properties of corn snacks produced using extrusion technology. While the control sample seems to be well expanded, the use of an enriching mixture caused the detrimental effect even at 10% level of addition (Table 6). This is typical for fiber-rich raw materials, which are used to replace starch or flour, such as defatted blackcurrant seeds [65], orange pomace [66] and several other off-products of industrial origin [47], although depending on the raw material and processing parameters opposite trends could also be found—e.g., in the cases of carrot pomace [67]. Gumul et al. [65] explained the decrease in expansion and increase in density of maize extrudates after the addition of the enrichment formulation by the fact that these samples contained more protein, fiber and sugar and less starch compared to the control.

Water-binding properties of the snacks were improved in comparison to the control, as both WEWAI and GEWAI significantly increased. It could be discussed if this could be caused by the larger amount of insoluble dietary fiber, as a drop in solubility could be observed only for the smallest addition level, and the extrudates with 30% of enriching mixture were characterized by significantly higher solubility than the control, but the values of WSI were not very reliable (Table 6).

Among the color parameters, L* varied a lot in the case of the control sample, revealing its highly heterogeneous character (analysis of variance could not be applied here, due to lack of homoscedascity, Table 6). The addition of a mixture of by-products to extrudates resulted in more homogeneous samples becoming slightly darker while elevating the level of addition. In the case of the control, the a* and b* color parameters were more uniform than L*. A significant increase in redness against greenness (a*) and yellowness against blueness (b*) could be observed after using a mixture of by-products, and the change in color was correlated with the level of addition. It seems obvious that as the content of red potato pulp increases in the formulation, the red color is more pronounced. Other components are probably also involved in the shift towards higher yellowness of the samples, but in this case, the differences between the extrudates with the addition of by-products are not obvious (Table 6).

Significant variability could also be observed for the cutting force of the extrudates, which was mainly due to high non-uniformity of the samples (Table 6). The lowest average values were calculated for extrudates with a share of 20% enriching mixture, but the other enriched samples did not seem to differ statistically from it. On the other hand, the control sample exhibited the largest value of cutting force together with the largest value of standard deviation, meaning that the extrudate strand was exhibiting different hardness at different cutting points (Table 6). Slightly decreasing average values (softer products) observed for snacks with a mixture of by-products, as compared to the control sample, were probably due to the enrichment in fiber, which caused a formation of more uniform cellular structure but also diminished the hardness of the cell walls of the extrudates.

In summary, the use of the applied by-product mixture will contribute to the production of snacks with reduced starch content and significant protein, fat, ash, and dietary fiber content. In addition, snacks enriched with this by-product mixture will be rich in polyphenols, phytosterols (mainly β-sitosterol, stigmasterol and campesterol), and tocopherols such as α- and γ-tocopherol. The use of by-products in the production of snacks corresponds to the zero-waste technology, which is one of the goals of sustainable development. It can therefore be said that the appropriate selection of by-products and extrusion process parameters will contribute to the creation of health-promoting snacks. The results show how health-promoting compounds can be introduced into food by using by-products, which is a global trend in current scientific research [68,69].

## 3. Materials and Methods

### 3.1. Materials

#### 3.1.1. Preparation of By-Products

The by-products (apple pomace, red potato pulp and sugar beet pulp in a ratio 1:1:1) were freeze-dried for 40 h using a Gamma 1-16 LSC freeze dryer (Martin Christ Gefriertrocknungsanlagen GmbH, Osterode am Harz, Germany) at −20 °C and a pressure of 0.1 mbar. After the freeze-drying process, the samples were ground using a Grindomix GM-200 laboratory grinder (Retsch GmbH&Co. KG., Haan, Germany) and used for further analysis.

#### 3.1.2. Preparation of Snacks

**Figure 2:**
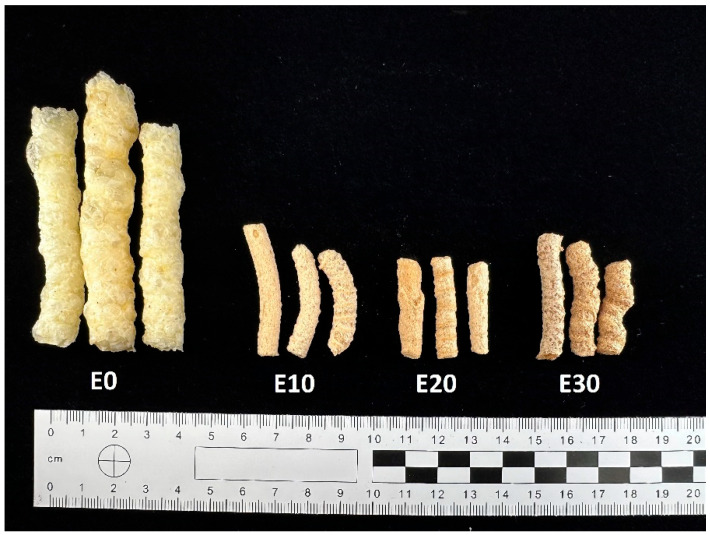
Snacks enriched with varying levels of a mixture of by-products.

Production of extrudates: extrudates containing 10%, 20%, 30%, by-products (E10; E20; E30) together with a control sample (E0) were prepared in a single-screw laboratory extruder. The cornmeal and by-products (apple pomace, red potato pulp, and beet pulp in a 1:1:1 ratio) were pre-ground to the appropriate size and then mixed. The moisture level in the components of the premix used for extrusion was set at 14%, and the particle sizes ranged from 600 to 950 μm. Part of the cornmeal was replaced with a premix in the amounts of 10%, 20%, 30%, resulting in plant-based snacks (E10–E30%). The extrusion was carried out using a single-screw extruder (Brabender, Duisburg, Germany). The screw speed was set at about 100 rpm. The diameter of the screw was 60 mm, and the extrusion process temperatures in different zones were as follows: zone I 140 °C/zone II 160 °C/zone III 130 °C (compression degree = 1:3). A matrix with two nozzles, each with a diameter of 3 mm, was used (Figure 2).

### 3.2. Methods

The following analyses were performed on raw materials (mixture of by-products) and snacks:

#### 3.2.1. Chemical Composition

The content of basic nutrients in the analyzed samples was determined by AOAC methods [70]. Protein (N × 6.25) was measured by the Kjeldahl method using the Kjeltec 2200 extraction device (Foss Analytical, Hillerød, Denmark). The total carbohydrate content was determined using the AOAC method no. 974.06. Fat content was measured using the Soxhlet method (AOAC method no. 953.38) using the Soxtec Avanti 2055 device (Foss Analytical, Hillerød, Denmark). The ash content was determined using the AOAC method No. 930.05. The content of total, soluble and insoluble dietary fiber (TDF, SDF, IDF) was determined by the AACCI method 32-07 [71], and the starch content was determined according to the standard 122/1 of ICC [72]. All the above measurements were taken in at least two repetitions.

#### 3.2.2. Analysis of Antioxidants and Anti-Radical Activities

Antioxidant compounds (polyphenols) and anti-radical activity in ethanol extracts were studied. A sample weighing 0.6 g was dissolved in 30 mL of 80% ethanol, shaken in the dark for 120 min (using an electric shaker, type WB22, Memmert, Schwabach, Germany), and then centrifuged at 4000 rpm for 15 min (MPW-350 centrifuge, MPW MED. Instruments, Warsaw, Poland). The supernatant was separated and stored at −20 °C for later analysis. The total polyphenol content (TPC) was determined by the spectrophotometric method with Folin–Ciocalteu reagent, according to Singleton et al. [73]. The contents of anthocyanins, phenolic acids and flavonols were determined using the methods of Mazza et al. [74], with modifications by Oomah et al. [75]. The methodology of El Hariri et al. [76] was applied to determine flavonoid content. Anti-radical potential was measured using the synthetic radical ABTS, according to Re et al. [77]. A diluted ethanol extract was combined with ABTS, shaken on a vortex mixer (WF2, Janke and Kunkel GmbH, Staufen im Breisgau, Germany), and its absorbance was measured using a spectrophotometer (Helios γ, 100–240 Thermo Spectronic, Runcorn, UK) at λ = 734 nm. The second reading was taken after 6 min at the same wavelength. The results were expressed in μmol TX/kg of dry matter (d.m.) Trolox equivalent (TEAC), where R2 = 0.999. Antioxidant activity in ethanol extracts was measured using 2,2-diphenyl-1-picrylhydrazyl (DPPH), according to Brand-Williams et al. [78]. Extracts (1 mL) were mixed with 4 mL of DPPH solution (0.012 g DPPH in 100 mL of ethanol). Absorbance was measured using a spectrophotometer at λ = 517 nm. Trolox (6-hydroxy2,5,7,8-tetramethylchromano-2-carboxylic acid) with R^2^ = 0.983 was used as a standard. Results are expressed in μmol/kg d.m. Trolox equivalent (TEAC).

#### 3.2.3. Determination of Tocopherols and Phytosterols

Determination of tocopherols and phytosterols was performed using gas chromatography. Tocopherols and phytosterols were determined by Hussain et al. [79], Oracz et al. [80] and Zhang et al. [81]. Samples were prepared by weighing 0.2 g of the sample (±0.0001 g) into a 20 mL vial. To this, 4 mL of freshly prepared saponification agent (3.9 mL of 2 M KOH in methanol and 0.5 mL of 10% ascorbic acid) was added. The vial was tightly closed, incubated at 85 °C for 40 min, and then cooled to room temperature. The contents were transferred to 30 mL centrifuge tubes containing 10 mL of hexane and 10 mL of saturated NaCl solution. The tubes were tightly closed, shaken for 10 min (175 rpm), and then centrifuged at 6000 rpm for 10 min. The top layer of hexane was collected, transferred to 20 mL vials, and evaporated under nitrogen. After drying, 1 mL of hexane was added, and the mixture was treated with ultrasound for 10 s. Samples were filtered through a nylon syringe filter (pore size 0.2–0.45 μm, ProSource Scientific, Calgary, AB, Canada). The analysis was performed using the Shimadzu GC 2010 Plus gas chromatograph with an FID detector (Shimadzu, Corp., Kyoto, Japan).

#### 3.2.4. Physical Parameters of Snacks

Density and coefficient of expansion were following Sokhey et al. [82]. Density determination was carried out by placing fragments of extrudate with a total weight of about 5 g in a cylinder with rapeseed and measuring the loss of their volume. The coefficient of expansion was calculated by dividing the average diameter of the extrudate by the diameter of the extruder nozzle (10 samples).

For determining the whole extrudate water absorption index (WEWAI), 1 g of whole extrudates was weighed and distilled water at room temperature was added. After wetting for 1 h, the extrudates were weighed, and the amount of water bound by them was obtained from the difference in mass. The result was then expressed per 1 g of product.

To measure the ground extrudate water absorption index (GEWAI) and solubility, 1 g of the product was ground, combined with 50 mL of distilled water and left at room temperature for 30 min. After this time, the sample was centrifuged for 5 min at 4000 rpm. Next, the supernatant was poured from above the sediment, and the amount of water absorbed by the sediment was determined for the calculation of GEWAI. The supernatant was poured into a Petri dish and dried at 50 °C for 24 h and the resulting mass was used to calculate the water solubility index [83].

The color of the samples was determined with a Konica Minolta CM-3500d spectrophotometer (Chiyoda, Japan) using a D65 illuminant and 10° observer angle. Five measurements were taken per sample and reported in the CIE Lab* system [84], with L* (lightness: 0 = black to 100 = white), a* (greenness to redness: negative to positive), and b* (blueness to yellowness: negative to positive).

#### 3.2.5. Statistical Analysis

Experimental data were subjected to analysis of variance (ANOVA) with Duncan’s multiple range test at a significance level of *p* ≤ 0.05 using Statistica software (version 8.0, StatSoft Inc., Tulsa, OK, USA). All analyses were performed at least in duplicate. The results are expressed as mean values ± standard deviation (SD). Pearson correlation coefficients were additionally calculated in Microsoft Excel for Microsoft 365 (Version 2403) using selected data series.

## 4. Conclusions

Snacks containing a mixture of by-products (apple pomace, red potato pulp, sugar beet pulp) can have a very beneficial dietary effect due to their high content of polyphenols, phenolic acids, anthocyanins, dietary fiber, tocopherols, especially α-tocopherol, and phytosterols, in particular: β-sitosterol, stigmasterol, and campesterol. In the production of plant-based snacks, a mixture in a 1:1:1 ratio of apple pomace, potato pulp, and beet pulp was used. The snacks were characterized by powerful antioxidant activities (measured with ABTS and DPPH) and desirable physical and functional properties (up to 20% level of addition). In addition, the above-mentioned by-product mixture increased the protein and ash content and contributed to a significant reduction in starch content in snacks. It can be suggested that snacks with a 20% share of by-product mixtures are recommended on an industrial scale due to their high contents of protein, dietary fiber, ash, antioxidants, tocopherols (especially α-tocopherol), phytosterols (β-sitosterol, stigmasterol, and campesterol), and acceptable physical and functional properties.

## Figures and Tables

**Table 1:** Chemical composition of the applied mixture of by-products (apple pomace, red potato pulp, sugar beet pulp in a ratio 1:1:1; before processing).

Component	Content
protein [g/100 g d.m.]	5.24 ± 0.20
fat [g/100 g d.m.]	1.47 ± 0.13
ash [g/100 g d.m.]	2.33 ± 0.12
total sugars [g/100 g d.m.]	26.00 ± 1.1
insoluble dietary fiber (IDF) [g/100 g d.m.]	23.60 ± 0.57
soluble dietary fiber (SDF) [g/100 g d.m.]	6.33 ± 0.17
total dietary fiber (TDF) [g/100 g d.m.]	29.93 ± 0.74
TPC [mg of catechin/g d.m.]	9.23 ± 0.09
phenolic acids [mg of ferulic acid/g d.m.]	2.38 ± 0.02
flavonols [mg of quercetin/g d.m.]	0.87 ± 0.01
flavonoids [mg of rutin/g d.m.]	5.06 ± 0.01
anthocyanins [mg of cyanidin 3-glucoside/g d.m.]	2.12 ± 0.01
ABTS [µMTX/kg d.m.]	172 ± 11
DPPH [µMTX/kg d.m.]	57.03 ± 0.01
δ-tocopherol [mg/100 g dm]	n.d. ^1^
α-tocopherol [mg/100 g dm]	1.04 ± 0.04
γ-tocopherol [mg/100 g dm]	0.33 ± 0.02
β-tocopherol [mg/100 g dm]	0.19 ± 0.01
cholesterol [mg/100 g dm]	0.16 ± 0.02
campesterol [mg/100 g dm]	0.89 ± 0.04
stigmasterol [mg/100 g dm]	1.34 ± 0.01
β-sitosterol (mg/100 g dm)	11.76 ± 0.01
δ-5-avenasterol [mg/100 g dm]	0.09 ± 0.01
δ-7-stigmasterol [mg/100 g dm]	n.d.
δ-7-avenasterol [mg/100 g dm]	n.d.

^1^ n.d—not detected.

**Table 2:** Chemical composition of snacks with a share of mixture of by-products (g/100 g d.m.).

Sample	Protein	Fat	Ash	IDF	SDF	TDF	Total Sugars	Starch
E0	4.56 ± 0.01 ^a1^	1.11 ± 0.01 ^a^	0.33 ± 0.01 ^a^	0.30 ± 0.01 ^a^	0.22 ± 0.05 ^a^	0.52 ± 0.04 ^a^	0.96 ± 0.04 ^a^	83.31 ± 0.08 ^d^
E10	4.42 ± 0.14 ^a^	1.10 ± 0.07 ^a^	0.57 ± 0.01 ^b^	1.88 ± 0.03 ^b^	0.81 ± 0.02 ^b^	2.69 ± 0.01 ^b^	2.82 ± 0.06 ^b^	78.22 ± 0.14 ^c^
E20	4.86 ± 0.04 ^b^	1.01 ± 0.03 ^a^	0.92 ± 0.01 ^c^	5.58 ± 0.15 ^c^	2.95 ± 0.02 ^c^	8.52 ± 0.13 ^c^	4.54 ± 0.04 ^c^	72.04 ± 0.07 ^b^
E30	5.35 ± 0.04 ^c^	0.98 ± 0.01 ^a^	1.24 ± 0.00 ^d^	7.41 ± 0.07 ^d^	4.26 ± 0.01 ^d^	11.67 ± 0.06 ^d^	5.75 ± 0.06 ^d^	64.28 ± 0.14 ^a^

^1^ Values denoted by the same letters in columns do not differ significantly (*p* < 0.05).

**Table 3:** Polyphenol compounds in snacks enriched with a mixture of by-products.

Sample	Anthocyanins [mg Cyanidin-3-Glycoside/100 g dm]	Total Phenolics TPC [mg Catechin/100 g d.m.]	Flavonoids [mg Rutin/100 g d.m.]	Content of Flavonols (mg Quercetin/100 g d.m.]	Content of Phenolic Acids [mg Ferulic Acid/100 g d.m.]
E0	n.d. ^1^	53.24 ± 0.01 ^a^	5.38 ± 0.07 ^a^	0.92 ± 0 ^a^	6.14 ± 0.72 ^a^
E10	1.05 ± 0.01 ^a2^	118.20 ± 1.32 ^b^	28.24 ± 1.2 ^b^	5.45 ± 0.74 ^b^	15.81 ± 0.01 ^b^
E20	7.42 ± 0.14 ^b^	215.14 ± 1.17 ^c^	68.53 ± 2.39 ^c^	16.87 ± 0.2 ^c^	34.24 ± 0.76 ^c^
E30	10.2 ± 0.09 ^c^	362.50 ± 1.98 ^d^	117.57 ± 1.02 ^d^	23.79 ± 0.09 ^d^	47.83 ± 1.52 ^d^

^1^ n.d—not detected; ^2^ Values denoted by the same letters in columns do not differ significantly (*p* < 0.05).

**Table 4:** Tocopherols in extrudates enriched with varying levels of a mixture of by-products.

Sample	δ-Tocopherol	β-Tocopherol	γ-Tocopherol	α-Tocopherol
E0	0.17 ± 0.01 ^a1^	n.d.	0.20 ± 0.01 ^a^	n.d.
E10	0.2 ± 0.03 ^a^	0.09 ± 0.04 ^a^	0.25 ± 0.01 ^b^	0.10 ± 0.03 ^a^
E20	0.21 ± 0.02 ^a^	0.15 ± 0.03 ^a^	0.27 ± 0.02 ^b^	0.18 ± 0.02 ^b^
E30	0.18 ± 0.01 ^a^	0.16 ± 0.02 ^a^	0.38 ± 0.04 ^c^	0.29 ± 0.03 ^c^

^1^ Values denoted by the same letters in columns do not differ significantly (*p* < 0.05), n.d.—not detected.

**Table 5:** Phytosterols in extrudates enriched with varying levels of a mixture of by-products.

Sample	Chole-Sterol	Campe-Sterol	Stigma-Sterol	β-Sitosterol	δ-5-Avena-Sterol	δ-7-Stigma-Sterol	δ-7-Avena-Sterol
E0	0.16 ± 0.01 ^a1^	0.10 ± 0.01 ^a^	0.30 ± 0.03 ^a^	0.74 ± 0.03 ^a^	0.03 ± 0.00 ^a^	1.24 ± 0.01 ^d^	0.21 ± 0.01 ^a^
E10	0.17 ± 0.00 ^a^	0.21 ± 0.01 ^b^	0.29 ± 0.01 ^a^	1.63 ± 0.11 ^b^	0.14 ± 0.00 ^b^	0.37 ± 0.00 ^a^	0.17 ± 0.00 ^a^
E20	0.25 ± 005 ^b^	0.48 ± 0.01 ^c^	0.42 ± 0.04 ^b^	4.57 ± 0.07 ^c^	0.42 ± 0.02 ^c^	0.55 ± 0.03 ^b^	0.21 ± 0.00 ^a^
E30	0.35 ± 0.01 ^c^	0.76 ± 0.13 ^d^	1.17 ± 0.05 ^c^	8.63 ± 0.05 ^d^	0.66 ± 0.03 ^d^	1.04 ± 0.00 ^c^	0.25 ± 0.03 ^a^

^1^ Values denoted by the same letters in columns do not differ significantly (*p* < 0.05).

**Table 6:** Physical properties of extrudates enriched with varying levels of a mixture of by-products (ER—expansion ratio, WEWAI—whole extrudate water absorption index, GEWAI—ground extrudate water absorption index, WSI—water solubility index, L*, a*, b*—color parameters).

Sample	Density [g/mL]	ER	WEWAI	GEWAI	WSI	L*	a*	b*	Cutting Force [N]
E0	0.05 ± 0.01 ^b1^	0.31 ± 0.04 ^b^	2.77 ± 0.24 ^a^	4.35 ± 0.33 ^a^	0.35 ± 0.03 ^b^	51.6 ± 11.8	1.36 ± 0.31 ^a^	11.6 ± 2.6 ^a^	24.4 ± 14.3
E10	0.06 ± 0.01 ^c^	0.24 ± 0.02 ^a^	3.69 ± 0.95 ^b^	5 ± 0.41 ^b^	0.28 ± 0.04 ^a^	58.3 ± 1.9	7.28 ± 0.96 ^b^	20.2 ± 2.3 ^b^	16.6 ± 6.1
E20	0.03 ± 0.01 ^a^	0.22 ± 0.04 ^a^	2.69 ± 0.65 ^a^	5.7 ± 0.04 ^c^	0.3 ± 0.05 ^ab^	54.1 ± 1.8	10.24 ± 0.33 ^c^	22.6 ± 0.6 ^b^	11.6 ± 7.5
E30	0.05 ± 0.01 ^b^	0.24 ± 0.05 ^a^	3.2 ± 0.25 ^b^	5.11 ± 0.06 ^b^	0.78 ± 0.71 ^c^	49.9 ± 4.5	12.58 ± 0.73 ^d^	23.6 ± 1.9 ^b^	12.4 ± 5.9

^1^ Values denoted by the same letters in columns do not differ significantly (*p* < 0.05).

## Data Availability

Data is contained within the article.

## Supplementary Materials

The following supporting information can be downloaded at: https://www.mdpi.com/article/10.3390/molecules31061037/s1.

## Abbreviations

The following abbreviations are used in this manuscript:

ABTS	2,2′-azino-bis (3-ethylbenzothiazoline-6-sulfonic acid)
DPPH	2,2-diphenyl-1-picrylhydrazyl (DPPH)
ER	expansion ratio
WEWAI	whole extrudate water absorption index
GEWAI	ground extrudate water absorption index
WSI	water solubility index
TDF	total dietary fiber
SDF	soluble dietary fiber
IDF	insoluble dietary fiber
