# Supplementary material for: Effect of Addition Levels of By-Product Mixture (Apple Pomace: Red Potato Pulp: Sugar Beet Pulp) on Phytochemical Profile, Antioxidant Activity and Physical Properties of Extruded Corn Snacks
Source: Molecules. 2026 Mar 20;31(6):1037. doi: 10.3390/molecules31061037 (PMC13029283; doi:10.3390/molecules31061037)
Supplement: Supplementary file 1 [file molecules-31-01037-s001.zip › molecules-4197368-supplementary.pdf]

Table S1. Correlation coefficients between analyzed parameters: T1 - Density; T2 - Expansion ratio; T3 - WAI-whole; T4 - WAI-ground; T5 - WSI; T6 - L*; T7 - a*; T8- b*; T9 - ABTS; T10 - DPPH; T11 - Protein; T12 - Fat; T13 - Ash; T14 - IDF; T15 - SDF; T16 - TDF; T17 - Total sugars; T18 - Starch; T19 - Anthocyanins; T20 - TPC; T21 - Flavonoids; T22 - Content of phenolic acids; T23 - δ-Tocopherol; T24 - β-Tocopherol; T25 - γ-Tocopherol; T26 - α-Tocopherol; T27 - Cholesterol; T28 - Campesterol; T29 - Stigma-sterol; T30 - β-Sitosterol; T31 - δ-5-avenasterol; T32 - δ-7-stigmasterol; T33 - δ-7-avenasterol.

	T1	T2	T3	T4	T5	T6	T7	T8	T9	T10	T11	T12	T13	T14	T15	T16	T17	T18	T19	T20	T21	T22	T23	T24	T25	T26	T27	T28	T29	T30	T31	T32	T33
T1	1.000																																
T2	0.352	1.000																															
T3	0.805	-0.262	1.000																														
T4	-0.651	-0.939	-0.078	1.000																													
T5	0.120	-0.103	0.080	0.000	1.000																												
T6	0.259	-0.333	0.566	0.208	-0.735	1.000																											
T7	-0.291	-0.876	0.194	0.796	0.566	-0.113	1.000																										
T8	-0.267	-0.952	0.287	0.856	0.402	0.092	0.979	1.000																									
T9	-0.403	-0.703	-0.050	0.689	0.722	-0.422	0.946	0.863	1.000																								
T10	-0.439	-0.696	-0.091	0.697	0.711	-0.438	0.938	0.853	0.999	1.000																							
T11	-0.323	-0.367	-0.202	0.379	0.892	-0.735	0.755	0.604	0.919	0.922	1.000																						
T12	0.531	0.651	0.214	-0.694	-0.686	0.499	-0.896	-0.801	-0.986	-0.992	-0.929	1.000																					
T13	-0.336	-0.711	0.023	0.670	0.742	-0.395	0.957	0.878	0.997	0.993	0.912	-0.971	1.000																				
T14	-0.444	-0.723	-0.077	0.722	0.688	-0.404	0.948	0.870	0.999	0.999	0.906	-0.990	0.993	1.000																			
T15	-0.427	-0.659	-0.106	0.661	0.741	-0.481	0.922	0.828	0.998	0.999	0.940	-0.993	0.991	0.996	1.000																		
T16	-0.438	-0.700	-0.087	0.700	0.709	-0.433	0.939	0.855	0.999	1.000	0.920	-0.992	0.993	0.999	0.998	1.000																	
T17	-0.348	-0.807	0.082	0.758	0.646	-0.259	0.989	0.938	0.984	0.979	0.842	-0.950	0.989	0.985	0.970	0.980	1.000																
T18	0.278	0.687	-0.064	-0.629	-0.775	0.405	-0.951	-0.868	-0.991	-0.985	-0.918	0.958	-0.998	-0.984	-0.985	-0.985	-0.983	1.000															
T19	-0.490	-0.653	-0.172	0.680	0.711	-0.495	0.907	0.812	0.992	0.996	0.936	-0.999	0.981	0.994	0.997	0.996	0.959	-0.971	1.000														
T20	-0.246	-0.624	0.052	0.564	0.825	-0.469	0.923	0.825	0.983	0.977	0.943	-0.952	0.991	0.973	0.981	0.977	0.963	-0.996	0.966	1.000													
T21	-0.290	-0.641	0.019	0.595	0.804	-0.466	0.929	0.833	0.991	0.986	0.942	-0.965	0.995	0.982	0.989	0.986	0.970	-0.998	0.976	0.999	1.000												
T22	-0.379	-0.704	-0.025	0.681	0.731	-0.416	0.950	0.868	1.000	0.998	0.918	-0.982	0.999	0.997	0.996	0.998	0.986	-0.994	0.989	0.987	0.993	1.000											
T23	-0.435	-0.832	0.131	0.855	-0.463	0.670	0.469	0.622	0.242	0.245	-0.146	-0.225	0.234	0.281	0.195	0.249	0.370	-0.191	0.211	0.108	0.137	0.237	1.000										
T24	-0.397	-0.919	0.124	0.874	0.460	-0.055	0.990	0.984	0.926	0.921	0.703	-0.889	0.930	0.935	0.901	0.923	0.974	-0.915	0.894	0.877	0.890	0.927	0.571	1.000									
T25	-0.052	-0.573	0.215	0.451	0.874	-0.431	0.892	0.796	0.932	0.918	0.906	-0.874	0.953	0.913	0.925	0.919	0.923	-0.971	0.896	0.981	0.971	0.941	0.024	0.824	1.000								
T26	-0.253	-0.737	0.126	0.663	0.739	-0.328	0.972	0.904	0.984	0.976	0.882	-0.942	0.995	0.978	0.973	0.977	0.991	-0.997	0.956	0.988	0.989	0.989	0.253	0.938	0.967	1.000							
T27	-0.293	-0.520	-0.069	0.495	0.863	-0.596	0.861	0.739	0.970	0.968	0.982	-0.958	0.971	0.959	0.979	0.967	0.924	-0.976	0.968	0.988	0.988	0.972	0.000	0.814	0.959	0.954	1.000						
T28	-0.325	-0.629	-0.026	0.597	0.801	-0.492	0.920	0.820	0.993	0.990	0.952	-0.974	0.994	0.985	0.994	0.989	0.966	-0.995	0.984	0.996	0.999	0.994	0.130	0.883	0.961	0.983	0.991	1.000					
T29	-0.003	-0.294	0.076	0.201	0.979	-0.669	0.718	0.571	0.845	0.835	0.947	-0.809	0.862	0.818	0.858	0.834	0.787	-0.887	0.831	0.922	0.907	0.853	-0.278	0.628	0.949	0.859	0.944	0.904	1.000				
T30	-0.269	-0.556	-0.020	0.516	0.855	-0.552	0.885	0.770	0.976	0.972	0.971	-0.956	0.980	0.964	0.981	0.971	0.939	-0.985	0.968	0.995	0.994	0.978	0.034	0.837	0.971	0.968	0.998	0.995	0.941	1.000			
T31	-0.362	-0.652	-0.046	0.630	0.774	-0.475	0.928	0.832	0.997	0.995	0.944	-0.982	0.996	0.992	0.997	0.995	0.973	-0.994	0.990	0.993	0.997	0.997	0.167	0.898	0.950	0.983	0.986	0.999	0.884	0.990	1.000		
T32	0.045	0.742	-0.484	-0.642	0.503	-0.877	-0.350	-0.535	-0.048	-0.036	0.347	-0.024	-0.066	-0.073	0.015	-0.041	-0.214	0.044	0.023	0.035	0.022	-0.052	-0.918	-0.421	0.045	-0.122	0.175	0.045	0.356	0.127	0.018	1.000	
T33	-0.324	0.000	-0.435	0.081	0.862	-0.940	0.446	0.254	0.706	0.716	0.923	-0.756	0.686	0.690	0.750	0.713	0.573	-0.694	0.757	0.742	0.741	0.701	-0.447	0.389	0.699	0.631	0.834	0.761	0.854	0.803	0.748	0.671	1.000
